# The Molecular Basis of Wnt/*β*-Catenin Signaling Pathways in Neurodegenerative Diseases

**DOI:** 10.1155/2023/9296092

**Published:** 2023-09-21

**Authors:** Ananya Anurag Anand, Misbah Khan, Monica V, Debasish Kar

**Affiliations:** ^1^Department of Applied Sciences, Indian Institute of Information Technology, Allahabad 211012, India; ^2^Department of Biotechnology, Ramaiah University of Applied Sciences, Bengaluru 560054, India

## Abstract

Defective Wnt signaling is found to be associated with various neurodegenerative diseases. In the canonical pathway, the Frizzled receptor (Fzd) and the lipoprotein receptor-related proteins 5/6 (LRP5/LRP6) create a seven-pass transmembrane receptor complex to which the Wnt ligands bind. This interaction causes the tumor suppressor adenomatous polyposis coli gene product (APC), casein kinase 1 (CK1), and GSK-3*β* (glycogen synthase kinase-3 beta) to be recruited by the scaffold protein Dishevelled (Dvl), which in turn deactivates the *β*-catenin destruction complex. This inactivation stops the destruction complex from phosphorylating *β*-catenin. As a result, *β*-catenin first builds up in the cytoplasm and then migrates into the nucleus, where it binds to the Lef/Tcf transcription factor to activate the transcription of more than 50 Wnt target genes, including those involved in cell growth, survival, differentiation, neurogenesis, and inflammation. The treatments that are currently available for neurodegenerative illnesses are most commonly not curative in nature but are only symptomatic. According to all available research, restoring Wnt/*β*-catenin signaling in the brains of patients with neurodegenerative disorders, particularly Alzheimer's and Parkinson's disease, would improve the condition of several patients with neurological disorders. The importance of Wnt activators and modulators in patients with such illnesses is to mainly restore rather than overstimulate the Wnt/*β*-catenin signaling, thereby reestablishing the equilibrium between Wnt-OFF and Wnt-ON states. In this review, we have tried to summarize the significance of the Wnt canonical pathway in the pathophysiology of certain neurodegenerative diseases, such as Alzheimer's disease, cerebral ischemia, Parkinson's disease, Huntington's disease, multiple sclerosis, and other similar diseases, and as to how can it be restored in these patients.

## 1. Introduction

The Wnt gene was first identified while studying the oncogenic mechanisms of the mouse mammary tumor virus (MMTV) [1]. The translated products of the Wnt gene are cysteine-rich, lipid-modified glycoproteins that are released into the extracellular environment [2]. The Wnt signaling pathway is of two types, namely, the canonical pathway and the noncanonical pathway, based on the different roles of *β*-catenin in the downstream processes. It is a key pathway that regulates cellular functions such as proliferation, translocation, differentiation, genetic stability, apoptosis, and stem cell renewal. This evolutionarily conserved pathway is also involved in the determination of cell polarity and cell fate during normal embryogenesis, in addition to maintaining tissue homeostasis in adults [3]. Owing to its pleiotropic effects and role in such essential processes, mutations or perturbations of the Wnt pathway are found to be associated with birth defects, cancer, and neurodegenerative disorders [4].

A deeper knowledge of the precise mechanism of Wnt signaling may offer insights into its influence on the onset and progression of several neurodegenerative disorders such as Alzheimer's disease, cerebral ischemia, Parkinson's disease, Huntington's disease, schizophrenia, multiple sclerosis, and amyotrophic lateral sclerosis [5]. It may also provide novel targets for therapeutic applications, making them an attractive target for the treatment of diseases [6]. In this regard, there has been an increase in research to find out novel strategies to target Wnt signaling in different neurodegenerative diseases. Various synthetic and natural molecules are being tested in the same regard. Efforts are also being made to find out efficient ways to restore Wnt signaling in different neurological diseases by creating a balance between Wnt-ON and Wnt-OFF states [7]. Thus, it becomes important to review the role of Wnt signaling in neurodegenerative diseases so as to come up with better treatment strategies for them. Hence, we have tried to bridge the gap between the available knowledge regarding the Wnt signaling pathways and their practical implications so that these can be modulated as per the disease condition.

## 2. Overview of Wnt Signaling Pathway

Wnt signaling pathway can be broadly classified into two main types: canonical Wnt/*β*-catenin pathway and noncanonical Wnt pathway.

### 2.1. Canonical Wnt Pathway

**Figure 1:**
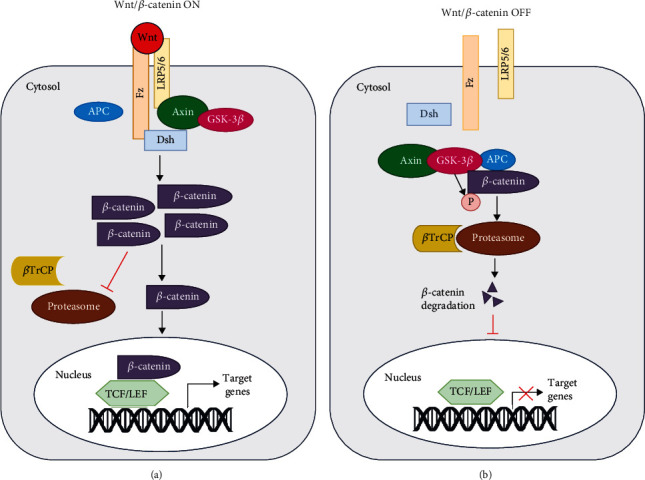
Wnt/*β*-catenin signaling. (a) When Wnt is present (Wnt-ON), it binds to Frizzled (fz) and its coreceptor lipoprotein receptor-related protein (LRP), thereby activating Dishevelled (Dsh). The destruction complex comprising axis inhibition protein (AXIN), adenomatous polyposis coli (APC), and glycogen synthase kinase-3*β* (GSK-3*β*) is disassembled. Hence, *β*-catenin is stabilized, translocates to the nucleus, binds to T cell factor/lymphoid enhancer-binding factor (TCF/LEF), and regulates gene expression. (b) When Wnt is absent, *β*-catenin is phosphorylated by the destruction complex and ubiquitinated by the E3 ligase *β*-transducin repeat-containing protein (*β*-TrCP), directing it towards proteasomal degradation. Hence, there is no transcription of downstream genes.

The canonical Wnt pathway is dependent on *β*-catenin, and therefore it is also referred to as the Wnt/*β*-catenin pathway. The characteristic feature of this pathway is the translocation of the adherens junction-associated protein, namely, *β*-catenin into the nucleus following its accumulation in the cytoplasm. It then binds to transcription factors like T cell factor (TCF) and lymphoid enhancer-binding factor (LEF) to activate the target genes (Figures 1(a) and 1(b)). The canonical pathway primarily regulates cell proliferation [8]. A subclass of Wnt proteins called Wnt1 proteins which includes Wnt1, Wnt2, Wnt3a, Wnt3, Wnt7a, Wnt8b, and Wnt10b are found to be involved in the Wnt/*β*-catenin signaling [9].

In the Wnt-ON state, secreted Wnt first binds to the extracellular cysteine-rich domain (CRD) at the N-terminal of the Frizzled (Fz) receptor. The Fz receptor is a seven-fold transmembrane-spanning protein that topologically resembles the G-protein-coupled receptors (GPCR) family and belongs to one of its subclasses [10]. Along with Fz, Wnt also binds to its coreceptor low-density lipoprotein-related protein 5/6 (LRP5/6). The signal is then transduced to Dishevelled (Dsh/Dvl), leading to its activation [11]. Dsh is a cytoplasmic phosphoprotein that is directly associated with Fz [12]. Activated Dsh triggers the disassembly of the destruction complex, which is comprised of Axin, adenomatous polyposis coli (APC), casein kinase 1 (CK1), and Ser/Thr kinase GSK-3*β* [13]. The activity of the GSK-3*β* enzyme is inhibited in the presence of Dsh. This interaction then activates a complex series of events that stabilize *β*-catenin by preventing its degradation, leading to its consequent accumulation in the cytoplasm [14]. The stabilized *β*-catenin now translocates into the nucleus, where it binds with the TCF/LEF transcription factor and controls the expression of target genes such as c-Myc, cyclin D-1, peroxisome proliferator-activated receptor-*γ* (PPAR*γ*), and metalloproteinases. These genes are involved in cell proliferation, survival, neurogenesis, and inflammation (Figure 1(a)) [15].

In the absence of Wnt (Wnt-OFF state), CK1 and GSK-3*β* of the destruction complex phosphorylate the N-terminal of cytoplasmic *β*-catenin. The phosphorylated *β*-catenin is ubiquitinated by the E3 ligase *β*-transducin repeat-containing protein (*β*-TrCP), thereby targeting it for ubiquitin-mediated proteasomal degradation. Hence, the nuclear translocation of *β*-catenin is blocked, and there is no expression of target genes (Figure 1(b)).

### 2.2. Noncanonical Wnt Pathway

**Figure 2:**
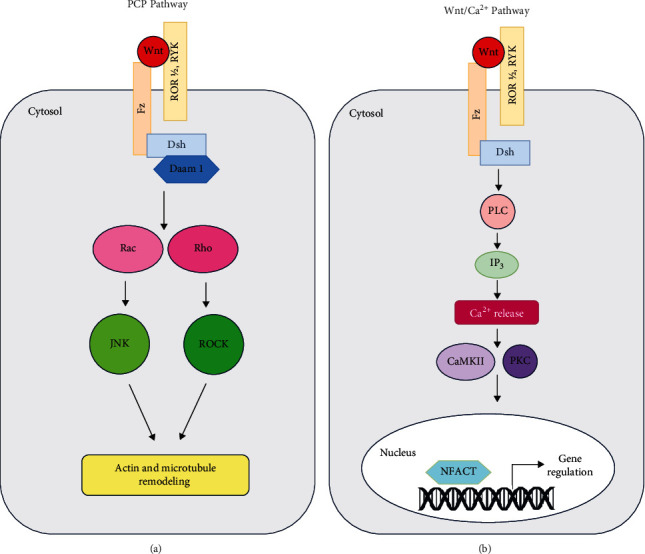
Noncanonical Wnt signaling. (a) In the planar cell polarity (PCP) pathway, Wnt binds to Fz independent of its LRP5/6 coreceptor and activates Dsh. The activated Dsh with the help of Daam1 activates Rac and Rho which in turn activate c-Jun-N-terminal kinase (JNK) and Rho kinase (ROCK), respectively. ROCK and JNK cause cytoskeletal changes that contribute to cell polarization. (b) In the Wnt/Ca^2+^ pathway, the signal is transduced by the binding of Wnt to Fz which activates Dsh. Dsh via phospholipase-C (PLC) activates inositol triphosphate (IP3) without the involvement of Daam1 like in PCP. This triggers the release of intracellular Ca^2+^ which in turn activates Ca^2+^/calmodulin-dependent protein kinase II (CamKII) and protein kinase C (PKC). This promotes the nuclear translocation of the nuclear factor of activated T cells (NFACT) and cAMP response element-binding protein (CREB) (not shown), which later binds to its target gene and regulates gene expression.

The noncanonical Wnt pathway is independent of *β*-catenin and TCF/LEF transcription factors and hence is known as the *β*-catenin-independent pathway. It is subdivided into two pathways: the planar cell polarity pathway (PCP) and the Wnt/Ca^2+^ pathway. The noncanonical pathway mainly determines cell polarity [16]. A subclass of Wnt proteins called Wnt5a proteins which include Wnt4, Wnt5a, and Wnt11 are involved in the noncanonical pathway. In this pathway, Wnts bind to Fz, which acts as a receptor, but instead of the LRP5/6 coreceptor, they bind to a variety of other coreceptors, namely, receptor tyrosine kinase (Ryk), collagen triple-helix repeat containing protein 1 (CTHRC1), and tyrosine-protein kinase transmembrane receptor Ror1/2. This is followed by the activation of Dsh by signal transduction (Figures 2(a) and 2(b)).

The PCP pathway is commonly known as the Wnt/PCP pathway or the Wnt/JNK pathway. In this pathway, activated Dsh transduces the signal to small G-protein molecules (GTPases) like Rho/Rac and activates them. Subsequently, c-Jun-N-terminal kinase (JNK) also gets activated. Because of this, reorganization of microtubules and actin filaments occurs (Figure 2(a)).

In the Wnt/Ca^2+^ pathway, interaction occurring between the Wnt ligand and Fz receptor stimulates the intracellular release of Ca^2+^ from the endoplasmic reticulum (ER) via phospholipase-c (PLC), which increases the intracellular level of Ca^2+^ and leads to decreasing the level of cyclic guanosine monophosphate (cGMP) [17]. This further activates Ca^2+^/calmodulin-dependent protein kinase II (CamKII) as well as protein kinase C (PKC) which are Ca^2+^-sensitive kinases. This leads to the nuclear translocation of the nuclear factor of activated T cells (NFACT) and cAMP response element-binding protein (CREB), which later binds to its target gene and regulates gene expression (Figure 2(b)).

Lately, it has been found that the transducers of the “canonical” Wnt pathway, i.e., GSK3 and *β*-catenin, and transducers of the “noncanonical” Wnt pathway, i.e., Wnt, RhoA, and RhoA kinase (RhoAK), can cooperate in order to control the expression of P-glycoprotein (Pgp) in the cells lying in the blood-brain barrier (BBB) [18]. Any disturbance in this cross-talk may downregulate Pgp and increase the delivery of Pgp substrates across the BBB, thereby interfering with the protection provided by the BBB to the CNS. In another recent study, it was found that members of the Wnt family of secreted glycoproteins are capable of regulating cell migration through distinct canonical and noncanonical signaling pathways [19]. Studies have shown that these pathways show opposing effects on cell migration. It was observed that in the nematode *Caenorhabditis elegans*, a switch from noncanonical to canonical signaling leads to the termination of long-range migration of QR neuroblast descendants. The noncanonical pathway acts via PIX-1/RhoGEF, while the canonical pathway directly activates the Slt–Robo component EVA-1/EVA1C and the Rho GTPase-activating protein RGA-9b/ARHGAP required for inhibiting migration. These studies show that the cross-talk between canonical and noncanonical Wnt signaling occurs via the antagonistic regulation of Rho GTPases which are responsible for cell migration.

### 2.3. Wnt Signaling in the Central Nervous System (CNS)

Wnt signaling plays a very important role in axon pathfinding, dendritic development, and synapse assembly by recruiting presynaptic and postsynaptic elements in both the central nervous system (CNS) and the peripheral nervous system (PNS) [20]. Wnt signaling affects both the structural and functional plasticity of synapses in the central nervous system as well as basal synaptic transmission. In spinal sensory neurons, Wnt3a along with Wnt7a promotes and directs growth cone remodeling and axon branching [21]. Field excitatory postsynaptic potentials (fEPSP) and synaptic NMDA-receptor currents are both amplified by Wnt5a, which makes it easier to induce excitatory long-term potentiation (LTP) [22]. In addition, NGF-dependent axonal development and branching also depend on Wnt5a. Other studies demonstrate that Wnt5a, via activating local PKC, can act as a key NGF downstream effector in the growth of sympathetic neurons [23]. Synapse development is aided by Wnt7a, which encourages the clustering of synapsin I and the expansion of the growth cone. Synapsin I clustering is found to be impaired in Wnt7a-null murine neurons [24]. Wnt proteins also play a significant role in dendritic development, maintenance, and neuronal activity. For instance, in cultured hippocampal neurons, Wnt2 promotes dendritic complexity, and Wnt7b increases dendritic length and forms complex branches at the time of dendritogenesis [25].

In a study, Wnt7a knockout mice were shown to possess fewer neural progenitor cells (NPCs), leading to an increase in the length of cell cycles, a decrease in reentry into the cell cycle, and impairment of neuronal differentiation [26]. This study indicates that Wnt7a plays a role in regulating proliferation and differentiation. In another study, a chronic infusion of Wnt7a was performed into the rat hippocampus, which led to an increase in the number of immature neurons [27]. In addition, immature neurons in Wnt7a knockout mice have been shown to exhibit a reduction in dendritic arborization. These studies indicate that Wnt7a plays multiple roles in adult hippocampal neurogenesis by controlling the development processes of newborn neurons.

Dsh enhances the differentiation of neuroblastoma 2A cell (N2A cell) and enables neuronal outgrowth. One of the three domains of Dsh is the N-terminal DVL domain (DIX) which is required for neuronal remodeling in N2A cells. Hence, DIX is crucial for N2A cells to differentiate [28]. Studies have also reported that Dsh enhances microtubule stability by inhibiting GSK-3*β* and restoring MAP-1B, which leads to the destabilization of axonal microtubules and shields them from nocodazole depolymerization [29]. Also, increased intracellular levels of *β*-catenin promote dendritic arborization, whereas sequestered endogenous *β*-catenin leads to a reduction in dendritic complexity.

In a recent study, the effect of the Wnt signaling pathway on neural progenitor cells isolated from the cerebral cortices of newborn mice was observed. It was found that the activation of Wnt canonical signaling reduced the expression of Hes1 and Hes5 genes in differentiating cell cultures, thereby interfering with the Notch pathway. This suggests that during the period of postnatal neural development, Wnt/*β*-catenin signaling enhances neurogenesis in progenitor cells [30].

## 3. Role of Wnt/*β*-Catenin Pathway in Different Neurodegenerative Diseases

An inflammatory response in the brain or spinal cord is referred to as neuroinflammation. It is associated with the production of cytokines, chemokines, reactive oxygen species (ROS), and disruption of the permeability of the blood-brain barrier (BBB). Endothelial cells, peripherally derived immune cells, and glial cells like microglia and astrocytes all contribute to the production of cytokines, chemokines, and ROS which are important mediators of neuroinflammation [31]. Wnt signaling also has immunomodulatory functions [32]. It regulates both adaptive and innate immune system responses at the time of inflammation [33]. It is established that proinflammatory activity is associated with a noncanonical Wnt pathway, while canonical Wnt signaling has anti-inflammatory effects.

Neuroinflammation is partially caused by the activation of the nuclear factor-kappa B (NF-*κ*B) pathway [34]. There is a close connection between the Wnt/*β*-catenin pathway and the NF-*κ*B pathway which becomes a crucial factor in both acute and chronic inflammation. The activity of the NF-*κ*B pathway is reduced by the activation of the Wnt/*β*-catenin pathway [35]. This interaction is in turn controlled by the GSK-3*β* protein, which stimulates *β*-catenin breakdown and positively modulates the NF-*κ*B pathway [36].

*β*-Catenin is a transcriptional activator that controls the expression of anti-inflammatory genes, thereby having an indirect influence on inflammatory reactions. PPAR*γ* is one such target gene of *β*-catenin. It exhibits an anti-inflammatory response by inhibiting the transcription of downstream genes of the NF-*κ*B pathway. In the rat hippocampus, PPAR*γ* activation has been found to be associated with lower GSK-3*β* activity, pointing to a possible interaction between PPAR*γ* and GSK-3*β* [37]. By studying the interaction between the Wnt/*β*-catenin pathway and neuroinflammation, we can find new treatment targets for neurological disorders. Thus, we will now discuss the role of the Wnt/*β*-catenin pathway in different neurodegenerative diseases, which are as follows.

### 3.1. Alzheimer's Disease

Two-thirds of dementia cases are associated with Alzheimer's disease (AD), which is characterized by gradual memory loss and cognitive impairment that makes performing daily tasks challenging. Wnt proteins are secreted glycoproteins that interact with the low-density lipoprotein receptor-related protein 5 (LRP5) or LRP6 and the extracellular cysteine-rich domain of the Frizzled (Fzd) receptor family to activate the canonical Wnt/*β*-catenin signaling pathway [38]. When Wnt binds to the Fzd/LRP5/6 receptor complex, LRP-Wnt-Fz complex is formed which leads to the inhibition of glycogen synthase kinase-3*β* (GSK-3*β*), and cytosolic *β*-catenin is stabilized. The expression of target genes is induced as a result of stabilized *β*-catenin translocating into the nucleus and interacting with TCF/LEF. But when LRP5/6 and Fzd bind to Dickkopf (DKK) and soluble Frizzled-related protein (sFRP), respectively, they block the formation of the LRP-Wnt-Fz complex in response to Wnts [39]. Synapses are directly impacted by decreased Wnt signaling. Deficient Wnt signaling may also have indirect impacts on synapses by interfering with the ability of microglia to survive.

According to a number of studies, the canonical Wnt antagonist DKK1 is upregulated in the brains of AD patients and AD mouse models. By interacting with LRP5/6 Wnt coreceptors, DKK1 prevents Frizzled and LRP5/6 from recognising Wnt proteins, hence inhibiting canonical Wnt signaling. The brains of AD patients show increased GSK-3*β* activity and decreased cytoplasmic *β*-catenin levels as a result of this suppression of Wnt signaling by DKK1 [40].

### 3.2. Cerebral Ischemia

During embryonic development, the Wnt/*β*-catenin signaling system regulates the establishment of the BBB and cerebrovascular development [41]. Clinically, some LRP6 genetic variations have been linked to an increased risk of ischemic stroke. Additionally, it has been noted that patients with acute ischemic stroke have greater plasma levels of DKK1 than healthy people. In cerebral ischemia (CI), it was discovered that elevated levels of DKK1 worsened the patient's condition by enhancing GSK-3*β* activity and obstructing the canonical Wnt pathway. The overexpression of DKK1 was observed in every case of Alzheimer's disease, Parkinson's disease, and cerebral ischemia [42]. In an experimental mouse with an ischemic stroke, lithium treatment showed a protective impact on BBB function. Furthermore, because BBB breakdown also occurs in leukaemia, toxic or metabolic encephalopathy, metastatic encephaloma, and other disorders involving BBB malfunction, it is important to look into the therapeutic potential of Wnt activators in these conditions [43].

### 3.3. Parkinson's Disease

Numerous genetic flaws that produce familial and idiopathic types of Parkinson's disease (PD) have been discovered over the past 20 years. Parallel to this, it has become clear that Wnt signaling pathways are essential for the adult brain's normal functioning and that these pathways are dysregulated in neurodegenerative diseases. Wnt signaling pathways are partially responsible for the disruption of cell biological processes in PD, and proteins encoded by PARK genes have been found to alter Wnt signaling. This raises the possibility of targeting Wnt signaling pathways in order to treat PD [44]. In Parkinson's disease (PD), strong GSK-3*β* activity and low Wnt/*β*-catenin signaling are correlated with high DKK1 expression [45]. Additionally, Wnt/*β*-catenin dysregulation has been linked to LRRK2 and Parkin mutations. Wnt signaling controls a variety of cellular processes, including the growth and maintenance of midbrain dopaminergic (mDA) neurons. Due to the fact that PD is brought on by the degradation of these neurons, they are of great interest in regenerative medicine [32]. In one of the studies, it has been found that in the 1-methyl-4-phenyl-1,2,3,6-tetrahydropyridine (MPTP) mouse model of PD, Wnt/*β*-catenin signaling along with glial cells regulate nigrostriatal DAergic neuronal health, protection, and regeneration. Therefore, it can be inferenced that Wnt/*β*-catenin signaling is capable of mediating a full neurorestorative programme in PD. Parkinson's disease (PD) has no known cure.

### 3.4. Huntington's Disease

Huntington's disease (HD) is a neurodegenerative disorder brought on by an increase in the number of CAG triplets in the gene that codes for the Huntington protein (Htt). It is characterized by selective neuronal death in the brain. According to recent data, the downregulation of the Wnt pathway in HD is correlated with lower transcription of Wnt prosurvival genes and consequently with higher levels of apoptosis. The Wnt canonical pathway contributes to HD progression by interfering with the turnover of *β*-catenin. Godin et al. specifically discovered an abnormal cytoplasmatic *β*-catenin build-up in the phosphorylated form that is unable to move in the nucleus and activate the transcription of target genes by using several *in vitro* and *in vivo* HD models [46]. In one of the studies, Inestrosa et al. carried out immune-coprecipitation tests in order to comprehend the main reason for *β*-catenin accumulation. The results showed that wild-type Htt functions as a scaffold protein, promoting *β*-catenin phosphorylation by the *β*-catenin destruction complex [47].

### 3.5. Schizophrenia

Schizophrenia is an extremely complex syndrome that involves dysregulation in cognition, behaviour, and emotion. According to recent research, schizophrenia's pathophysiology may be influenced by immunological dysfunction and central nervous system (CNS) inflammation. Neuroinflammation in schizophrenia is correlated with disruption of the Wnt/*β*-catenin pathway [48].

Despite the fact that no Wnt ligands have been discovered to be genes of interest in genome-wide association studies (GWAS) or other linkage studies, Wnt1 has been reported to be upregulated in the schizophrenic brain, and it has been found that some genes are linked to disease susceptibility and are essential elements of the Wnt signaling pathway [49]. The discovery of TCF4 (T cell factor 4) has been found to be significantly linked with schizophrenia [50]. TCF4, along with the *β*-catenin coactivator, functions as one of the major transcriptional mediators of the canonical Wnt signaling [51]. TCF4 haploinsufficiency causes the Pitt-Hopkins syndrome, which involves severe mental retardation as one of its characteristics [52]. Additionally, it has been noted that TCF4 overexpression in the brain results in memory problems and, more significantly, prepulse inhibition problems, which are neurophysiological correlates of schizophrenia and other psychiatric illnesses.

Genetic tests have also linked DKK proteins to schizophrenia. Extracellular DKK proteins compete with Wnts for binding to LRP5/6 receptors and therefore damage the Fzd-LRP5/6 complex that starts Wnt/*β*-catenin signaling. In genome investigations of mutations linked to schizophrenia, DKK1, DKK3, and DKK4 have all been identified as genes of relevance, along with the DKK1 coreceptor KREMEN1 [53]. In one of the studies, gene expression profiles of hiPSC neurons in schizophrenia patients showed altered expression of various components of the Wnt signaling pathways. Thus, the role of Wnt in schizophrenia needs to be understood in greater depth to see how modulating Wnt signaling can help treat these patients [54].

### 3.6. Multiple Sclerosis

Among other signaling pathways, the Wnt/*β*-catenin/Tcf signaling pathway has been recognised as a crucial factor in the control of oligodendrocyte development and remyelination [55]. Numerous investigations have suggested that Wnt molecules hinder oligodendrocyte maturation. Studies have proven that Wnt3a, a member of the Wnt signaling family, is linked to oncogenesis and a number of developmental processes, such as the control of cell fate during embryogenesis, that might have implications in multiple sclerosis as well [56].

*β*-Catenin is a component of the cadherin complex and has the ability to control cell-cell adhesion and cell migration. Any imbalance in the level of *β*-catenin may lead to serious problems that can further trigger the onset of multiple sclerosis [57]. In one of the studies, myelin thickness was assessed using g-ratio analysis. *β*-Catenin-overexpressing mice showed a considerably lower g-ratio when compared to wild-type mice [58].

Wnt signaling has also been demonstrated to contribute to the emergence of persistent pain in multiple sclerosis. One study found that Wnt signaling pathways are activated abnormally in the SCDH of EAE mouse models, which may contribute to the development of chronic pain associated with the disease [59]. Thus, one of the important pathways governing myelination, remyelination, and chronic pain in schizophrenia patients is the Wnt signaling pathway.

### 3.7. Amyotrophic Lateral Sclerosis

Studies related to amyotrophic lateral sclerosis (ALS) have demonstrated a correlation between glial cell growth and aberrant activation of the Wnt/*β*-catenin signaling pathway. The degeneration of motor neurons in ALS is caused by the significant buildup of *β*-catenin in those cells [60]. In ALS, astrogliosis is influenced by Wnt/*β*-catenin signaling activation. The conversion of microglia to a proinflammatory phenotype is also encouraged by the activation of Wnt/*β*-catenin signaling. Wnt/*β*-catenin signaling has also been found to be associated with delayed maturation of regenerated oligodendrocytes. It has been found that Wnt/*β*-catenin signaling encourages oligodendrogliogenesis and the restoration of degenerative myelin structure in preexisting oligodendrocytes [61].

The malfunctioning of the complete motor unit, from axon terminal denervation to motor neuron death, appears to be mediated by Wnts, Wnt receptors, and other essential elements of the Wnt pathways [62]. Another example of the unique function of Wnts is provided by the aberrant expression of Wnt ligands in ALS. Firstly, aberrant Wnt/*β*-catenin signaling activation causes glial cell growth and neuronal degeneration. The Wnt/PCP routes (Wnt/Ca^2+^ and Wnt/*β*-catenin signaling pathways) are interdependent, which makes this signal network more complex. Secondly, the MuSK (muscle-specific kinase) receptor in SOD1-G93A mice has been the subject of a growing body of research, and this research suggests that gene therapy is crucial for treating ALS. Moreover, the diverse LRP4 antibody patterns across various ethnic groups point to considerable individual variations in the ALS disease processes [63]. We will get a better knowledge of the mechanisms behind ALS and will be better equipped to explore innovative therapeutic options if the linkage between ALS and Wnt signaling is fully deciphered.

## 4. Strategies for Targeting the Wnt/*β*-Catenin Signaling in Various Neurodegenerative Diseases

**Figure 3:**
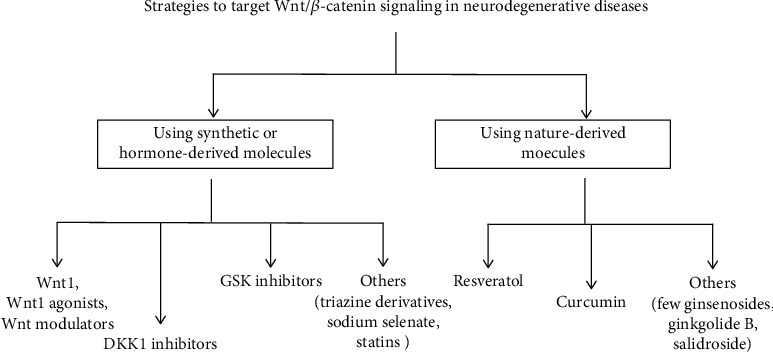
Strategies for targeting Wnt/*β*-catenin signaling in neurodegenerative diseases. The signaling pathway followed by Wnt/*β*-catenin can be targeted by using both synthetic and nature-derived molecules in order to treat several neurodegenerative disorders.

There are currently a few methods for halting or slowing the progression of PD, AD, and ALS [64]. Although many medicines have been studied in recent years, very few Wnt modulators have been reported, and information on the neuroprotective ability of Wnt-related compounds is still scarce [65]. Many of the drugs that are being studied in preclinical and clinical settings for cancer therapy that act as Wnt agonists, such as DKK inhibitors and GSK inhibitors, may be possible candidates for devising novel therapeutic strategies against neurodegeneration [66]. The various strategies that can be used to target the Wnt/*β*-catenin pathway so as to treat various neurodegenerative diseases are summarized in Figure 3.

### 4.1. Synthetic or Hormone-Derived Molecules Targeting Upstream Events of the Wnt/*β*-Catenin Signaling

#### 4.1.1. Wnt1, Wnt1 Agonists, and Wnt Modulators

The communication between glial cells and neurons is mediated by Wnt1 and Wnt1 agonists and is responsible for providing a critical neuroprotective mechanism against oxidative stress, growth factor deprivation, and inflammation. Microglial and astrocytic cells in the brain are responsible for the primary secretion of Wnt1 and Wnt1 agonists [67]. These two have been reported to exhibit neuroprotective properties against DA neuron-specific toxins, such as 6-hydroxydopamine when administered exogenously. Interestingly they are also shown to improve motor symptomatology in cellular and animal models of Parkinson's disease [68].

A few years ago, it was discovered that the hormone atrial natriuretic peptide (ANP), which is produced by the heart and is essential for maintaining cardiovascular homeostasis, has antiproliferative effects on colorectal cancer cells by influencing Wnt/*β*-catenin signaling. It is interesting to note that natriuretic hormone acts as a Wnt agonist in DA neuron-like cultures, enhancing signaling where the system is essentially not activated [69]. On the other hand, ANP is responsible for the suppression of the Wnt/*β*-catenin pathway in colorectal cancer cells (where this pathway is otherwise constitutively active). ANP essentially acts as a Wnt modulator that can restore the equilibrium between the Wnt-OFF and Wnt-ON states to successfully reverse aberrant Wnt signaling in a number of clinical situations. Endogenous ANP appears to be crucial for brain development, synaptic transmission, and neuroprotection in the CNS, where it is abundantly generated and mostly secreted by glial cells.

#### 4.1.2. DKK1 Inhibitors

Dickkopf (DKK) proteins are LRP5/6 antagonists and act as inhibitors of the Wnt/*β*-catenin pathway. The most likely method by which DKK1 inhibits Wnt signaling seems to be the breakdown of the Fzd/LRP6 complex. As a negative regulator of the Wnt/*β*-catenin pathway, DKK1 is one of the most studied inhibitors to modulate Wnt in neurodegenerative diseases, including AD. DKK1-neutralizing antibodies can counteract the effects of acute exposure to A*β* oligomers. A*β* oligomers have been demonstrated to trigger DKK1 expression and synaptic site loss in mouse brain slices from AD patients [70]. Recent research has also shown that inhibiting the DKK1 transmembrane receptor, KREMEN1 (Krm1), using miR-431 prevents A*β*-mediated synapse loss in corticohippocampal cells taken from transgenic AD mice [71].

#### 4.1.3. GSK Inhibitors

Glycogen synthase kinase-3*β* (GSK-3*β*), one of the five enzymes able to phosphorylate glycogen synthase, was initially isolated from skeletal muscle in 1980. By regulating a number of signaling pathways, including the Wnt/*β*-catenin pathway, GSK-3*β* governs several important cellular processes in the brain [72]. It is commonly regarded as a therapeutic target of interest since dysregulation of this kinase is relevant to the pathogenesis of several neurological illnesses, including AD and PD. GSK-3*β* is a component of the destruction complex in the Wnt-OFF state, where it quickly phosphorylates extra *β*-catenin before the ubiquitin-proteasome pathway degrades it.

On the other hand, suppression of GSK-3*β* results in the stability of *β*-catenin and activates signaling (Wnt-ON state). The GSK inhibitors SB216763 and LiCl (lithium chloride) have been shown to activate Wnt/*β*-catenin signaling in a cellular model of Parkinson's disease [73]. It has also been demonstrated that valproic acid (VPA), a first-line antiepileptic and mood stabiliser used in clinics for almost 50 years, directly inhibits GSK. VPA may also improve cognitive function in AD. VPA is responsible for the activation of the Wnt/*β*-catenin pathway and has recently been found to improve the process of neurogenesis in the adult hippocampus of the corresponding mouse model of Alzheimer's. Lithium and VPA have been evaluated for their efficacy in treating neurological diseases in clinical trials. However, their efficacy was not related directly to Wnt/*β*-catenin [74]. Lithium was used in the clinical trials either solely as a carbonate salt or in combination with valproate, GTA, or riluzole. Likewise, VPA was assessed either alone or in combination with levocarnitine, lithium, and quetiapine. MK-801, an NMDA (N-methyl-D-aspartate) receptor, also known as dizocilpine, is another compound which has been found to be responsible for indirect inhibition of GSK-3*β*. In one of the studies, MK-801 has been shown to increase Wnt3a levels in hemiparkinsonian rats to inhibit GSK-3*β*, which in turn activates Wnt/*β*-catenin signaling and boosts hippocampus neurogenesis [75].

#### 4.1.4. Other Synthetic or Hormone-Derived Molecules That Directly or Indirectly Target the Signaling

*(1) Triazine Derivatives (Cholinesterase Inhibitors)*. Triazines have received great attention as cholinesterase inhibitors. The formation of A*β* fibrils is promoted by a type-B carboxylesterase enzyme known as acetylcholinesterase (AChE). AChE is responsible for the hydrolysis of acetylcholine (Ach) along with some other choline esters and is present in the synaptic cleft. This interaction between A*β* fibrils and AChE is responsible for the production of the AChE-A*β* complex which increases A*β*-dependent neurotoxicity [76]. Subsequent studies have revealed that there is a connection between the Wnt signal transduction pathway and AChE-A*β* neurotoxicity which leads to the assumption that cholinesterase inhibitors should have a positive impact on the Wnt/*β*-catenin pathway. Cholinesterase inhibitors have already been established as one of the symptomatic methods for the treatment of Alzheimer's disease [77]. Owing to their potent antianxiety, antidepressant, and antiepileptic functions, specifically in the case of Alzheimer's, where they exert a neuroprotective function, the spectrum of triazines as neuropharmacological agents is continuously expanding.

*(2) Sodium Selenate*. Selenium is a crucial trace element for the brain, playing a role in many CNS processes, such as memory and cognition [78]. There is growing evidence that dietary selenium consumption is negatively correlated with mortality in AD patients. In the form of several selenoproteins, selenium performs a variety of biological roles. When compared to other selenium compounds, sodium selenate considerably increases phosphatase activity by acting as a selective agonist for protein phosphatases of type 2A (PP2A). PP2A is a heterotrimeric serine-threonine phosphatase that controls a number of physiological activities via protein dephosphorylation [79]. It performs a variety of tasks, including dephosphorylating phosphorylated APP and inactive *β*-catenin [80]. Sodium selenate has been shown to greatly increase the activity of PP2A and suppress the symptoms of the illness in a mouse model of AD [81]. Selenate-treated mice are shown to have lower levels of APP phosphorylation, resulting in lesser APP breakage and A*β* plaque development [82].

*(3) Statins*. Recent studies have reported that statins, a class of drugs used to treat hyperlipidemia in clinical settings, exhibit neuroprotective characteristics and are therapeutically beneficial for neurological illnesses like Parkinson's disease (PD), Alzheimer's disease (AD), and all forms of dementia [83]. Simvastatin in particular, developed by the Merck company and authorised for medical use in 1992, has been connected to a significant drop in the prevalence of dementia and Parkinson's disease (PD). In both *in vitro* and *in vivo* conditions, simvastatin can also encourage neurogenesis [84]. Although statins, particularly simvastatin, have been investigated in phases 1-4 of clinical trials for Parkinson's disease and Alzheimer's disease, they do not have a specific or direct relation to Wnt/*β*-catenin signaling [85].

### 4.2. Nature-Derived Molecules That Target Wnt/*β*-Catenin Signaling

Several natural compounds have been identified as modulators of Wnt signaling [86]. Numerous studies have been conducted to show the effectiveness of these modulators in treating neurological diseases. Natural substances targeting Wnt signaling are being researched in preclinical studies as potential treatments for brain disease and have been demonstrated to induce neuronal differentiation and exhibit neuroprotective properties [87]. These include ginsenosides, salidroside, resveratrol, and curcumin. The weak bioavailability of these biomolecules and their infrequent transport to the brain have limited their effectiveness in treating neurodegenerative diseases [88]. In recent years, cutting-edge delivery methods that might increase their neuroavailability and, consequently, their neuroprotective effect, are being developed [89]. Three natural substances—resveratrol, curcumin, and ginkgolides—are presently undergoing clinical studies for the treatment of a range of neurological disorders [90]. However, only a few studies using cellular and animal models of PD and AD have investigated the effect of these natural chemicals on Wnt/*β*-catenin signaling.

#### 4.2.1. Resveratrol

A polyphenolic phytoalexin called resveratrol is present in a variety of foods, including blueberries, grapes, peanuts, and wines. Resveratrol has been shown to have antioxidant, cardioprotective, neuroprotective, and anticancer properties [91]. Resveratrol is indicated to be a potential treatment for age-related cognitive disorders, including Alzheimer's disease. SAMP8 (senescence-accelerated prone mice P8) is being employed as a mouse model for Alzheimer's disease and ageing [92]. Enhanced cognitive abilities and a relatively lower degree of neurodegeneration were observed in SAMP8 mice treated with dietary resveratrol [93]. The Wnt/*β*-catenin pathway has recently been explained further by Palomera-Avalos et al. [86], which supports the theory that resveratrol's antioxidant and anti-inflammatory effects also have an impact on mitochondrial functioning and inflammatory processes. In particular, they showed that following a high-fat diet, the Wnt/*β*-catenin signaling pathway was activated, leading to metabolic stress in the SAMP8 mouse model [94].

#### 4.2.2. Curcumin

Curcumin, a polyphenol, is obtained from the rhizome of the ginger family flowering plant *Curcuma longa*. Traditional treatments like Ayurvedic and Chinese traditional medicine frequently employ *Curcuma longa* extract. Curcumin has intriguing biological properties, like anti-inflammatory, antioxidant, and neuroprotective [95]. It can inhibit the production of Wnt antagonist DKK1 and promote the expression of Wnt proteins. The ability of curcumin to decrease the production of GSK-3*β* and induce the expression of *β*-catenin to activate Wnt/*β*-catenin signaling has also been established. Activation of the Wnt/*β*-catenin pathway using curcumin can be made possible by using modern delivery strategies that enhance neuroavailability, as in the case of curcumin nanoparticles [96].

#### 4.2.3. Other Natural Compounds

According to one of the studies, the principal bioactive component of *Panax ginseng*, ginsenoside Rg1, may exert neuroprotective effects in PD models both *in vitro* and *in vivo* through the Wnt/*β*-catenin pathway and has been recommended as a potential treatment strategy. Another ginsenoside, Rb1, was shown to be a robust inhibitor of synuclein fibrillation and toxicity. In addition, it has been reported to exhibit a high capacity to disintegrate fibrils and prevent the polymerization of *α*-synuclein *in vitro* [97].

*Ginkgo biloba* extract, one of the ingredients in Chinese herbal medicines, has been shown to enhance memory along with spatial learning ability in a transgenic mouse model of Alzheimer's [98]. It has also been shown to reduce the symptoms of AD and age-related dementia. *Ginkgo biloba* extract and ginkgolide B, one of its primary components, have been reported to activate the Wnt/*β*-catenin pathway, thereby promoting the development of neurons in neural stem cells [99]. Interestingly, ginkgolide B may pass the BBB, which makes it more prudent as a possible treatment for brain injuries or neurodegenerative diseases.

Salidroside, a phytobioactive compound, is a phenol glycoside obtained from the antifatigue herb *Rhodiola rosea* and has been proposed as a potent neuroprotective agent for the treatment of neurodegenerative diseases, especially PD. It is enriched with several pharmacological properties like being an antioxidative, anticancerous, antifatigue, and anti-inflammatory agent [100]. A recent study conducted in a rat model of PD has revealed that this compound can shield against oxidative stress caused by Wnt/*β*-catenin pathway activation. A labdane diterpenoid, andrographolide, is known to activate the Wnt/catenin pathway and drive transcription of Wnt target genes through direct inhibition of GSK-3*β* by employing a substrate-competitive mechanism. It is an annual herb which has been used as a natural anti-inflammatory compound for thousands of years in China and India with no reported side effects [101].

## 5. Restoring Wnt/*β*-Catenin Signaling

Since various pathogenic pathways in neurological illnesses significantly reduce Wnt/*β*-catenin signaling, restoring Wnt/*β*-catenin signaling can be one of the potential strategies with respect to the development of innovative neurological therapeutics. Wnt signaling controls a variety of biological functions, including cell survival. In addition to being essential for neuronal survival and neurogenesis, Wnt signaling in the brain also controls synaptic plasticity, BBB integrity, and function. Many more such activities are performed by Wnt signaling. For instance, in the case of AD, in the adult hippocampus, synaptic integrity depends on Wnt signaling. Loss of synapses, long-term memory, and deficiencies in synaptic plasticity are brought on by DKK1. So, therefore, reactivating the Wnt pathway can reverse memory deficits and synapse loss.

Most of the neuronal processes that are damaged in neurological disorders would be improved if Wnt signaling was restored in the brains of individuals with brain diseases, especially PD and AD [102]. The treatments for neurodegenerative illnesses that are present in the market are primarily symptomatic and not curative. Thus, there is a need for a curative treatment of such diseases [103]. In this regard, targeting Wnt signaling seems to be a promising approach. It has been demonstrated that a number of Wnt pathway elements, including GSK-3*β*, *β*-catenin, APC, TCF7L2, and FZD3, are connected to schizophrenia as well [5]. According to one study, the antipsychotic loxapine medication improves the Wnt signaling components in fibroblasts derived from induced pluripotent stem cells (iPSCs) from schizophrenia patients [54]. Therefore, we must try to come up with novel curative strategies against neurological diseases that involve the restoration of the Wnt signaling pathway. Also, the therapeutic application of Wnt activators/modulators should aim to restore rather than overstimulate Wnt signaling, thereby reestablishing the balance between the Wnt-OFF state and the Wnt-ON state.

## 6. Conclusion and Future Directions

The development of novel treatment approaches for treating neurodegenerative illnesses may result from a better understanding of the molecular processes underlying their pathophysiology. There is mounting evidence that the onset and progression of neurodegenerative disorders are significantly influenced by changes in Wnt canonical signaling. Overall, we think that the beginning and progression of the majority of neurodegenerative illnesses may be influenced by the loss or gain of function of the Wnt/*β*-catenin pathway. The evidence from the literature points to the consensus that restoring the Wnt canonical pathway to normal levels has a neuroprotective impact. Therefore, a viable therapeutic approach to prevent the onset of neurodegenerative illnesses might involve the stimulation of Wnt signaling through the exogenous injection of Wnt agonists or its suppression through the use of Wnt antagonists. With the exception of some naturally occurring chemicals like ginkgolide B, there are not many specific blood-brain barrier- (BBB-) permeant Wnt activators which could serve as a potential treatment for PD, AD, or other neurological disorders. The limited brain bioavailability of these compounds may reduce their usefulness. However, the advent of innovative delivery methods, such as drug-loaded nanoparticles or mucoadhesive formulations, established with the intention of enhancing medication neuroavailability, may be the solution to this issue. Additionally, nose-to-brain delivery is a noninvasive technique that enables the transfer of complicated medications to the CNS directly or via nanosized carriers, evading the BBB. To further circumvent the BBB and treat neurodegenerative diseases, exploring hydrogel-based biomaterials or nanoengineered drug-releasing implants could also be a good option. Finally, there are certain issues that need to be addressed regarding the safety of targeting a signal transduction system that is essential for tissue homeostasis and repair. Overstimulating Wnt/*β*-catenin signaling to accomplish neuroprotection and neurorepair might be tumorigenic because incorrect stimulation of this signaling has been linked to specific forms of cancer. Therefore, the reestablishment of balance between the Wnt-OFF and ON states in patients with neurodegenerative disorders can be achieved by therapeutic administration of Wnt activators or modulators which function in such a manner as to just restore the normal functioning of the Wnt/*β*-catenin pathway instead of overstimulating it by any means.

## Data Availability

No data are associated with this article.
